# Subsequent chemotherapy with paclitaxel plus cetuximab-based chemotherapy following immune checkpoint inhibitor in recurrent or metastatic squamous cell carcinoma of the head and neck

**DOI:** 10.3389/fonc.2023.1221352

**Published:** 2023-11-21

**Authors:** Hideki Tanaka, Tomohiro Enokida, Susumu Okano, Takao Fujisawa, Nobukazu Tanaka, Naohiro Takeshita, Ryutaro Onaga, Yuta Hoshi, Akihisa Wada, Masanobu Sato, Yuri Ueda, Makoto Tahara

**Affiliations:** ^1^ Department of Head and Neck Medical Oncology, National Cancer Center Hospital East, Kashiwa, Japan; ^2^ Department of Otorhinolaryngology-Head and Neck Surgery, Tokyo Medical University, Tokyo, Japan; ^3^ Department of Otorhinolaryngology, Jikei University School of Medicine, Tokyo, Japan; ^4^ Department of Head and Neck Surgery, Tokyo Medical and Dental University, Tokyo, Japan; ^5^ Department of Otorhinolaryngology, Nagoya University Graduate School of Medicine, Nagoya, Japan; ^6^ Department of Otorhinolaryngology, Graduate School of Medical Sciences, Kyushu University, Fukuoka, Japan

**Keywords:** pembrolizumab, subsequent chemotherapy, paclitaxel, cetuximab, carboplatin, recurrent/metastasis squamous cell carcinoma of the head and neck, immune checkpoint inhibitor

## Abstract

**Background:**

Immune checkpoint inhibitors (ICIs) are essential in treating recurrent/metastatic squamous cell carcinoma of the head and neck (R/M SCCHN). However, the overall response rate (ORR) is limited to 10-20%, and subsequent chemotherapy is critical to maximizing the subjects’ prognosis.

**Methods:**

We retrospectively reviewed 59 patients with R/M SCCHN treated with paclitaxel+cetuximab (PE)-based chemotherapy (PCE, paclitaxel+carboplatin+cetuximab; or PTX+Cmab, paclitaxel+cetuximab) following disease progression after either pembrolizumab or nivolumab monotherapy.

**Results:**

Of 59 patients, 15 were treated with pembrolizumab, with an ORR of 13.3%, and the remaining 44 with nivolumab, with an ORR of 11.4%. All patients in the pembrolizumab cohort had platinum-sensitive disease. Following ICI treatment, 19 patients were treated with PCE and the remaining 40 with PTX+Cmab. PE-based chemotherapy induced favorable and prompt tumor shrinkage even in cases where ICI was not effective, with a median change in the summed dimensions of target lesions of -43.4%, resulting in an ORR of 62.7%. Median time to response was 1.8 months. The patients in the pembrolizumab cohort appeared to have a numerically higher response rate than those receiving nivolumab (80.0% vs. 56.8%). For the 59 patients, progression-free survival and overall survival, calculated from the initiation of PE-based chemotherapy, were 4.6 months and 17.1 months, respectively. Grade ≥3 adverse events occurred in 40.7%, and no treatment-related death was observed.

**Conclusion:**

PE-based chemotherapy following ICI is encouraging for its robust antitumor efficacy in R/M SCCHN.

## Introduction

The treatment of recurrent/metastatic squamous cell carcinoma of the head and neck (R/M SCCHN) was significantly advanced with introduction of the anti-EGFR antibody cetuximab (Cmab) in 2005 (1), and further improved with the advent of immune checkpoint inhibitors (ICIs) (2, 3). Among these, the anti-programmed cell death protein 1 (PD-1) antibodies pembrolizumab and nivolumab have become the standard systemic therapy for R/M SCCHN. Although these agents provide a durable response and favorable safety profile, overall response rates (ORR) to them as monotherapy are generally modest (around 10-20%) (2, 3). Moreover, the KEYNOTE-048 study revealed a reverse relationship between relatively short progression-free survival (PFS) and longer PFS2 or overall survival (OS) with pembrolizumab monotherapy, indicating the importance of subsequent therapy following ICI (4). Several plausible explanations for the robust efficacy of subsequent therapy have been offered, including the additive or synergistic immunologic interplay between the two, based on the long half-life of ICI as prior therapy (5). To date, however, no standardized regimen following ICI has yet been established.

Against this background, taxane-containing regimens are now considered the most promising therapeutic options. Conventionally, paclitaxel (PTX) plus Cmab-based (PE-based) chemotherapy, such as the combination of PTX, carboplatin (CBDCA), and Cmab (PCE), as well as the combination of PTX and Cmab (PTX+Cmab), have played a critical role in R/M SCCHN (6, 7). Indeed, these combinations are reported to show robust efficacy (8–10)—primarily PTX+Cmab after nivolumab—probably due to the augmentation of anti-tumor immunity by both agents (11, 12). Further, the recent subgroup analysis of the KEYNOTE-048 study revealed that, in a post-pembrolizumab monotherapy setting, non-taxane-containing 2nd line therapy might be associated with unsatisfactory PFS2 compared with taxane-containing therapy, again indicating a positive interaction between PE-based chemotherapy and ICI. However, no study has specifically focused on regimens following 1st line pembrolizumab monotherapy.

Here, we report the overall efficacy and safety of PE-based chemotherapy following ICI therapy. We then drill down into regimens by preceding ICI treatment line and discuss their expected roles as a component of sequential therapy in this patient population.

## Materials and methods

### Patient

We retrospectively reviewed the patients who had received PE-based chemotherapy (PCE or PTX+Cmab) following ICI (pembrolizumab or nivolumab) monotherapy at National Cancer Center East between May 2017 and April 2022. Other inclusion criteria included (1) histologically proven SCC; (2) primary tumor location in the larynx, oral cavity, oropharynx, or hypopharynx; and (3) tumor recurrence or initial distant metastasis. To extract patients with these conditions, we used a computer-managed search system based on the prescribed regimens, and we then collected their clinical data from each medical record. Platinum-sensitive was defined as disease progression or recurrence after 6 months from the end of platinum chemotherapy, and platinum-refractory was defined as occurring within 6 months.

### Treatment

For pembrolizumab monotherapy, all patients were treated with a three-week cycle of pembrolizumab 200 mg/m2 on day 1. Nivolumab was administered to patients at 240 mg/m2 on day 1 in a two-week cycle.

For the PCE regimen, patients were treated with a three-week cycle of PTX with 100 mg/m2 and CBDCA with the area under the concentration-time curve (AUC) of 2.5 on days 1 and 8. For the PTX+Cmab regimen, patients were treated with a four-week cycle of PTX 80 mg/m2 on days 1, 8, and 15 of each cycle. Cmab was given in an initial dose of 400 mg/m2 followed by 250 mg/m2 weekly until disease progression or unacceptable toxicities in both regimens. In the PCE regimen, patients were treated with Cmab alone as maintenance therapy after six cycles of the PCE combination phase. Dose modification was allowed in accordance with the patient’s systematic status and treatment-related adverse events. In the PTX+Cmab regimen, patients were treated with PTX until disease progression or unacceptable toxicities occurred. Computed tomography (CT) or magnetic resonance imaging (MRI) to evaluate treatment efficacy was carried out approximately every 1–3 months, or sooner if the physician in charge deemed it necessary.

### Evaluation of efficacy and statistical analysis

Clinical outcomes of the PE-based chemotherapy are presented as overall response rate (ORR), disease control rate (DCR), time to response (TTP), progression-free survival (PFS), overall survival (OS), and adverse events (AEs). Clinical tumor response was evaluated according to the Response Evaluation Criteria in Solid Tumors (RECIST) ver. 1.1 guidelines, including complete response (CR), partial response (PR), stable disease (SD), and progressive disease (PD). ORR and DCR were defined as a proportion of CR and PR, and of CR, PR, and SD, respectively. AEs were evaluated by CTCAE ver. 5.0. Time to response was measured from the first day of PE-based chemotherapy administration until the first date of evaluation by imaging in the patients who appear PR or CR by PE-based chemotherapy. PFS was measured from the first day of PE-based chemotherapy administration until the date of tumor progression or death from any cause. OS was calculated from the first day of PE-based chemotherapy to the date of death from any cause. Further, PFS2 were measured from the first day of ICI monotherapy administration until the date of tumor progression by subsequent chemotherapy, herein PCE or PTX+Cmab, or death from any cause. Moreover, time from the first day of ICI monotherapy administration until date of death from any cause was defined as OSici. The Kaplan–Meier method was used to assess these prognostic data. Univariate analysis was performed using log-rank test. All analyses were performed using SPSS ver. 28 (IBM Corp., Armonk, NY, USA) with a data cut-off of August 31, 2022.

## Results

### Patient characteristics

Fifty-nine patients (pembrolizumab as prior ICI in fifteen patients and nivolumab as prior ICI in forty-four patients) treated with PE-based chemotherapy following ICI monotherapy were identified in this study (**Supplementary Figure 1**). Baseline characteristics of the entire population are presented in **Table 1**. Most patients were male (78.0%), and median age was 63 years. The most common primary tumor site was the oral cavity (37.3%), followed by the oropharynx (32.2%). The most common disease distribution at the initiation of PE-based chemotherapy was local/regional metastasis only (42.4%), and platinum-refractory disease was observed in 54.2%. Nineteen patients (32.2%) were treated with PCE and the remaining forty (67.8%) with PTX+Cmab. Further, the different backgrounds of the pembrolizumab and nivolumab cohorts are shown in **Supplementary Table 1**. All patients in the pembrolizumab cohort harbored platinum-sensitive and PD-L1-positive (≥1 in CPS) disease, versus only the minority of patients in the nivolumab cohort (100% vs. 27.3%, p<0.01). PCE tended to be selected for patients who were treated with pembrolizumab as prior therapy, compared with nivolumab (73.3% vs. 32.2%, p<0.01).

**Table 1:** Patient characteristics.

Characteristic	Number of patients, (%)
**Median Age**, years [range]	63 [19-79]
Gender	
Male	46 (78)
Female	13 (22)
ECOG performance status score	
0	27 (45.8)
1	22 (37.3)
2	6 (10.2)
Unknown	4 (6.8)
Primary tumor site	
Oral cavity	22 (37.3)
Hypopharynx	13 (22)
Larynx	5 (8.5)
Oropharynx	19 (32.2)
p16 (+)	6 (31.6)
p16 (-)	7 (36.8)
p16 unknown	6 (31.6)
Smoking status	
Current or former	48 (81.4)
Never	11 (18.6)
Disease distribution	
Local/regional metastasis only	25 (42.4)
Distant metastasis only	19 (32.2)
Local/regional and distant metastasis	15 (25.4)
Platinum sensitivity	
Sensitive	27 (45.8)
Refractory	32 (54.2)
PD-L1 status (CPS)	
**1-19**	8 (13.6)
**20-**	7 (11.9)
**Unknown**	44 (74.6)
Prior immunotherapy regimens	
Pembrolizumab	15 (25.4)
Nivolumab	44 (74.6)
Efficacy by ICI	
Complete response (CR)	1 (1.7)
Partial response (PR)	6 (10.2)
Stable disease	12 (20.3)
Progressive disease	38 (64.4)
Not evaluable	2 (3.4)
**ORR by prior ICI** (%)	11.9
**Median PFS by prior ICI**, month [95%CI]	2.3 [1.8-4.1]
Number of previous lines of systemic therapy before ICI for R/M SCCHN	
0	48 (81.4)
1	10 (16.9)
≥2	1 (1.7)
Regimen of PE-based chemotherapy	
PCE	19 (32.2)
PTX+Cmab	40 (67.8)

ECOG, Eastern Cooperative Oncology Group; CI, confidence interval; CPS, combined positive score; ORR, overall response rate; PFS, progression-free survival; ICI, immune check point inhibitor; R/M HNSCC, recurrent/metastatic squamous cell carcinoma of the head and neck; PTX+Cmab, paclitaxel and cetuximab; PCE, paclitaxel+carboplatin+cetuximab. Platinum-sensitive was defined as disease progression or recurrence after 6 months from the end of platinum chemotherapy, and platinum-refractory was defined as occurring within 6 months.; ORR, proportion of CR+PR.

### PE-based chemotherapy following ICI

**Figure 1:**
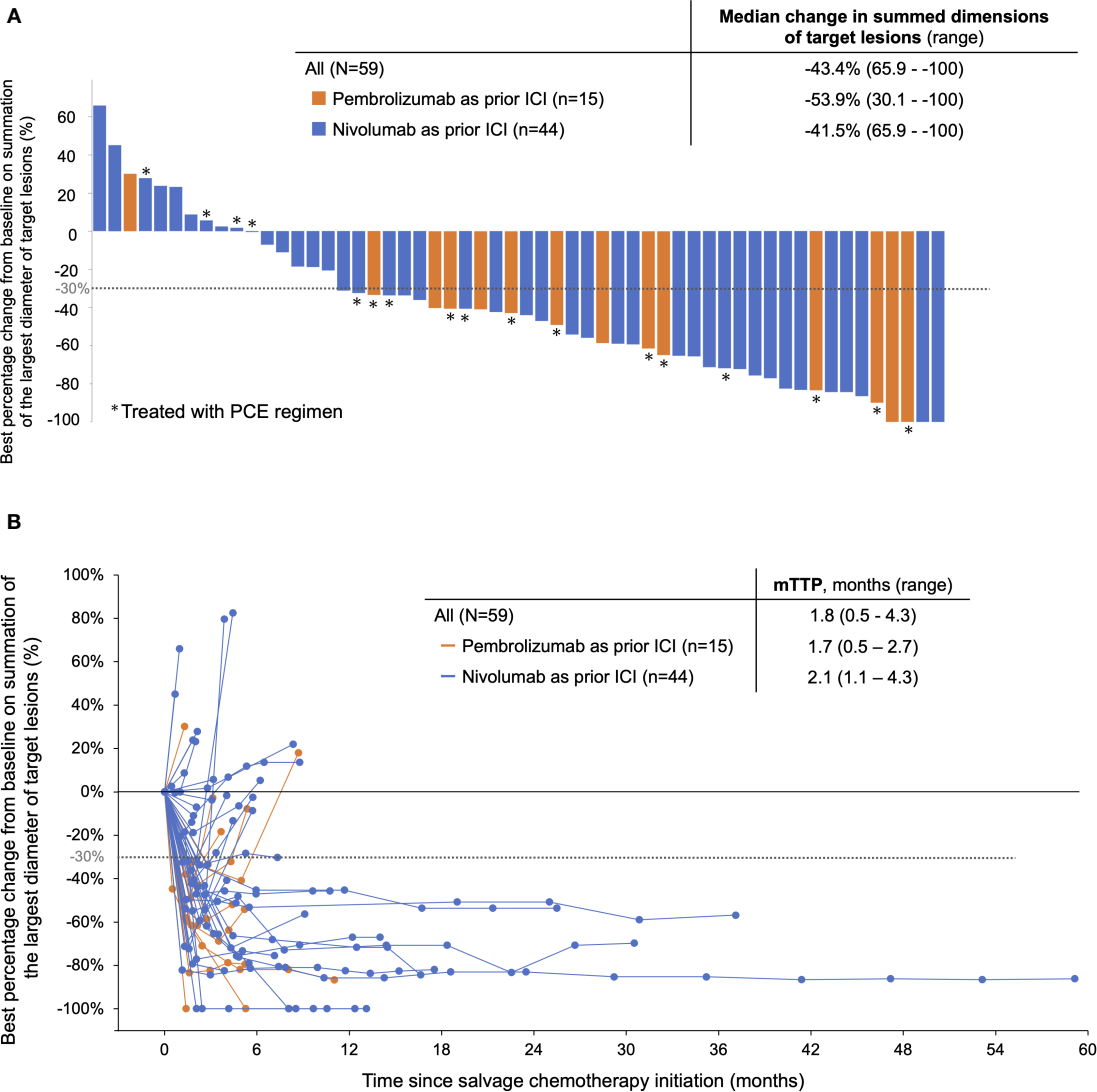
Response of patients treated with PE-based chemotherapy following ICI monotherapy. **(A)** Best response to PE-based chemotherapy by RECIST ver.1.1. **(B)** Spider plot showing the change in the sum of tumor diameters based on RECIST during PE-based chemotherapy. PCE, paclitaxel+carboplatin+cetuximab; PTX+Cmab, paclitaxel+cetuximab, TTP, time to response. Note: ICI, pembrolizumab or nivolumab monotherapy; PE-based chemotherapy, PCE or PTX+Cmab.

**Figure 2:**
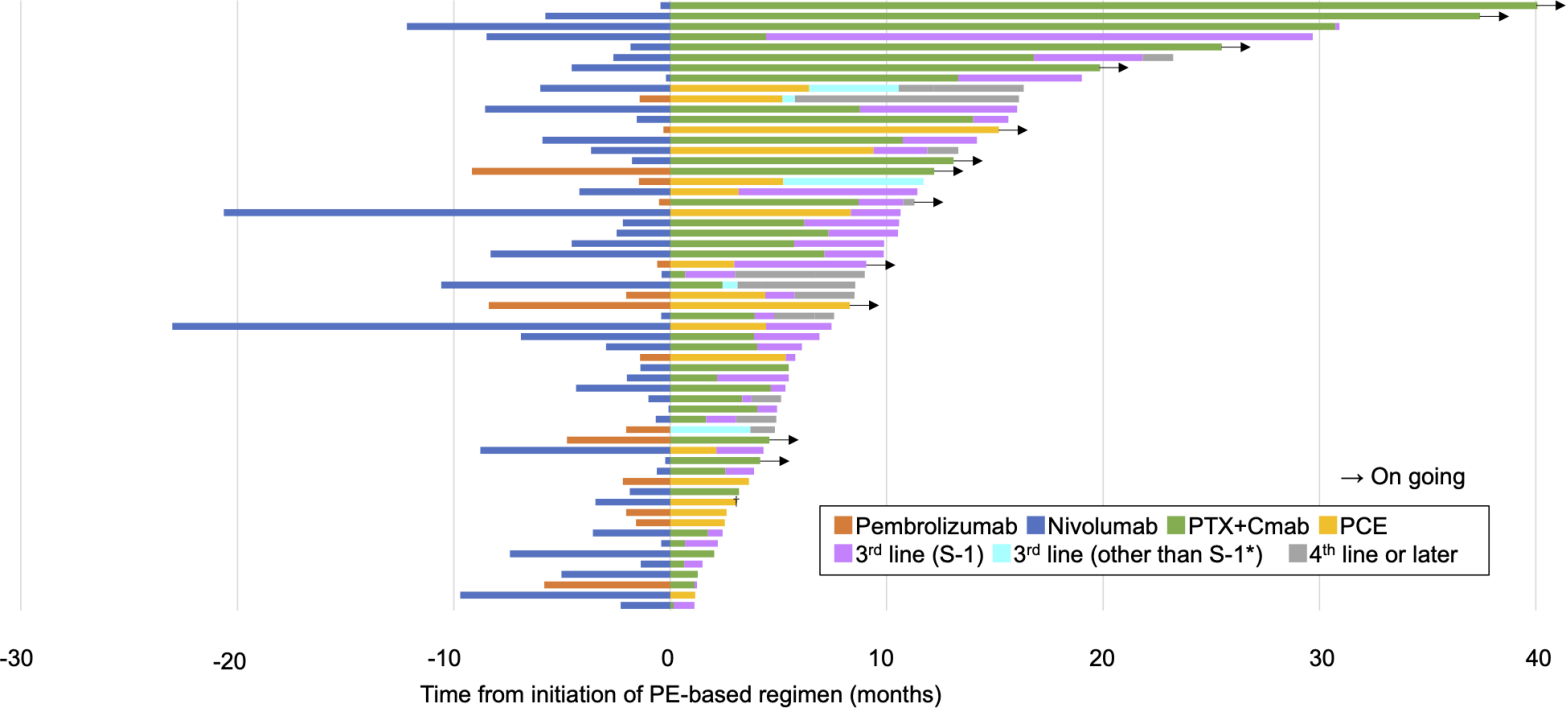
Swimmer plot showing the overall clinical course. PTX+Cmab, paclitaxel+cetuximab; PCE, paclitaxel+carboplatin+cetuximab. *nivolumab (n=3), 5-FU+carboplatin+cetuximab (n=1) and docetaxel (n=1). †One patient received surgery for oligoprogressive disease in a locoregional lesion.

**Figure 3:**
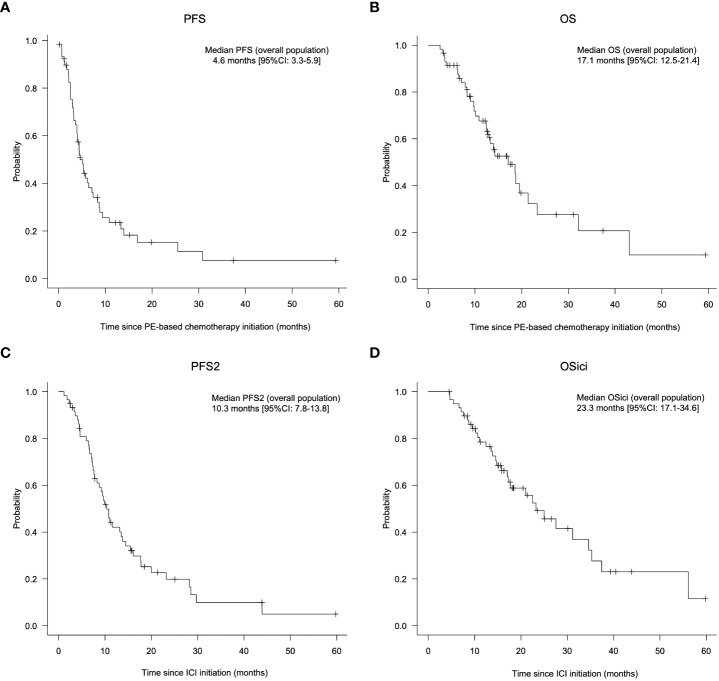
Prognosis of patients treated with PE-based chemotherapy following ICI monotherapy. A, B: Progression-free survival **(A)** and overall survival **(B)** from the initiation of PE-based chemotherapy in all patients. (C, D) Progression-free survival 2 (PFS 2) **(C)** and overall survival (OSici) **(D)** from initiation of immune checkpoint inhibitors in all patients. PCE, paclitaxel+carboplatin+cetuximab; PTX+Cmab, paclitaxel+cetuximab; PFS, progression-free survival; OS, overall survival; PFS2, progression-free survival 2; CI, confidence interval; ICI, immune checkpoint inhibitor. Note: PE-based chemotherapy, PCE or PTX+Cmab; ICI, pembrolizumab or nivolumab; OSici, overall survival from initiation of immune checkpoint inhibitors.

Regarding the efficacy of PE-based chemotherapy in the entire population, CR, PR, SD, and PD were obtained in 3 (5.1%), 33 (57.6%), 7 (11.9%), and 13 (22.0%) patients, respectively, with an ORR and DCR of 62.7% and 74.6% (**Table 2**). The median change in the summed dimensions of target lesions was -43.4% (range: 65.9% to -100%), and the ORRs of PCE and PTX+Cmab were very similar (63.2% in PCE and 62.5% in PE) (**Figure 1A** and **Table 2**). In addition, tumor response was rapid, evidenced by a median time to response of 1.8 months (range: 0.5-4.3) (**Figure 1B**). A large majority of responders to the paclitaxel plus cetuximab-based chemotherapy had no response to immune checkpoint inhibitor monotherapy: of the 38 patients classified with disease progression following immune checkpoint inhibitor, 25 responded to the PE-based chemotherapy (**Supplementary Figure 2**). Also, the duration of ICI treatment success did not appear to be associated with PE-based chemotherapy duration: even patients who experienced rapid tumor progression in the ICI phase achieved long disease control with PE-based chemotherapy following failure (**Figure 2**). In the PE-based chemotherapy phase, median length of hospitalization throughout the treatment period was 0 days (range: 0-33), and 44 patients (74.6%) received all treatment with the regimen on an outpatient basis. Fifty patients experienced disease progression on PE-based chemotherapy as of data-cutoff, and 41 (82.0%) switched to subsequent treatment (**Supplementary Figure 3**). With a median follow-up of 12.4 months from the initiation of PE-based chemotherapy, PFS and OS were 4.6 months (95%CI: 3.3-5.9) and 17.1 months (95%CI: 12.1-22.1), respectively (**Figures 3A, B**). These results clearly indicate a significant advantage in PFS and OS (p < 0.01, both) when compared to the outcomes observed with other regimens besides PE-based chemotherapy (n = 55) for subsequent chemotherapy following ICI treatment during the same observation period (**Supplementary Figure 4**). In addition, from the initiation of ICI, PFS2 and OSici were 10.3 months (95%CI: 7.8-13.8) and 23.3 months (95%CI: 17.1-34.6), respectively (**Figures 3C, D**).

**Table 2:** Clinical response by PE-based chemotherapy.

Response	Number of patients, (%)		
	Total (N=59)	PCE (n=19)	PTX+Cmab (n=40)
Complete response (CR)	3 (5.1)	1 (5.3)	2 (5.0)
Partial response (PR)	34 (57.6)	11 (57.9)	23 (57.5)
Stable disease (SD)	7 (11.9)	3(15.8)	4 (10.0)
Progressive disease	13 (22.0)	3 (15.8)	10 (25.0)
Not evaluable	2 (3.4)	1 (5.3)	1 (2.5)
**ORR** (%)	37 (62.7)	12 (63.2)	25 (62.5)
**DCR** (%)	44 (74.6)	15 (78.9)	29 (72.7)

ORR, overall response rate; DCR, disease control rate; PTX+Cmab, paclitaxel+cetuximab; PCE, paclitaxel+carboplatin+cetuximab. ORR, proportion of CR+PR; DCR, proportion of CR+PR+SD.

When we looked at the efficacy of PE-based chemotherapy according to prior ICI, the reduction in target lesion and ORR for PE-based chemotherapy appeared more prominent in the pembrolizumab cohort than in the nivolumab cohort (median change in summed dimensions of the target lesions: -53.9% vs. -41.5% in **Figure 1A**; ORR, 80.0% vs. 56.8% in **Table 3**). Kaplan Meier curves for PFS and OS from the initiation of PE-based chemotherapy are shown in **Supplementary Figure 5** (PFS: 5.2 months [95% CI: 3.2-7.0] and OS: 17.1 months [95% CI: 3.4-not available] in the pembrolizumab cohort) and **Supplementary Figure 6** (PFS: 4.6 months [95% CI: 2.0-7.2] and OS: 18.6 months [95% CI: 11.7-25.5] in the nivolumab cohort), respectively. In pembrolizumab cohort, PFS2 and OSici were 7.6 months (95% CI: 6.0-9.2) and 21.0 months (95%CI: 6.3- 33.3), respectively (**Supplementary Figures 5C and D**). Whereas, in the nivolumab cohort, PFS2 and OSici were 11.6 months (95% CI: 8.5-14.7) and 25.0 months (95% CI: 17.6-32.4), respectively (**Supplementary Figure 6C and D**).

**Table 3:** Clinical response by PE-based chemotherapy according to prior ICI therapy.

Response	Number of patients, (%)	
	Pembrolizumab (n=15)	Nivolumab (n=44)
Complete response (CR)	2 (13.3)	1 (6.7)
Partial response (PR)	10 (66.7)	24 (54.5)
Stable disease (SD)	0 (0)	7 (15.9)
Progressive disease (PD)	2 (13.3)	11 (25.0)
Not evaluable (NE)	1 (6.7)	1 (2.3)
**ORR** (%)	12 (80.0)	25 (56.8)
**DCR** (%)	12 (80.0)	32 (72.7)

ORR, overall response rate; DCR, disease control rate; ORR, proportion of CR+PR; DCR, proportion of CR+PR+SD.

In **Supplementary Figure 7** and **Supplementary Table 2**, We focused on the platinum sensitivity and compared the efficacy of PE-based chemotherapy between platinum sensitive and platinum refractory patients. All platinum refractory patients were treated with nivolumab as prior immune therapy and none were treated with PCE regimen as subsequent chemotherapy after ICI. There was a difference between platinum sensitive and refractory patients in the number of previous lines of systemic therapy before ICI for R/M SCCHN, however no difference was founf in PFS, OS, PFS2, and OSici.

Acute toxicities experienced during the PE-based chemotherapy are listed in **Table 4**. Grade 3 or worse AEs were observed in 24 patients (40.7%), of which leukopenia (11.7%), neutropenia (6.9%), mucositis (6.8%), and pneumonia (6.8%) were most common. There was no treatment-related death throughout treatment.

**Table 4:** Selected toxicity during PE-based chemotherapy.

	Number of patients, (%)	
	Any Grade	Grade 3/4
Hematological toxicity		
Leukopenia	21 (35.6)	7 (11.7)
Neutropenia	16 (27.1)	5 (6.9)
Febrile neutropenia	0 (0)	0 (0)
Anemia	16 (27.1)	3 (5.1)
Thrombocytopenia	7 (11.9)	0 (0)
Non-hematological toxicity		
AST elevation	13 (22)	0 (0)
ALT elevation	9 (15.3)	0 (0)
Enterocolitis	3 (5.1)	1 (1.7)
Diarrhea	3 (5.1)	0 (0)
Mucositis	15 (25.4)	4 (6.8)
Nausea	6 (10.2)	0 (0)
Fatigue	21 (35.6)	1 (1.7)
Skin reactions^*^	45 (76.3)	3 (5.1)
Peripheral neuropathy	15 (25.4)	0 (0)
Pneumonia	6 (10.2)	4 (6.8)
Hypomagnesemia	5 (8.5)	1 (1.7)
Hypokalemia	2 (3.4)	1 (1.7)
Infusion reaction/anaphylaxis	6 (10.2)	2 (3.4)
**Total**	58 (98.3)	24 (40.7)

ALT, alanine aminotransferase; AST, aspartate amino transferase. ^*^Skin reactions were coded with the use of preferred terms from the Medical Dictionary for Regulatory Activities. These terms include acne pustular, acne, cellulitis, dermatitis acneiform, dry skin, erythema, nail-bed infection, nail-bed inflammation, nail disorder, nail infection, paronychia, pruritus, and rash. Graded according to common toxicity criteria for adverse events version 5.0.

In **Table 5**, the univariate analysis showed that number of lines of ICI were prognostic factors for PFS. OS did not differ significantly but tended to be worse later in the line of ICI administration. In addition, univariate analysis showed a significant difference between PS 0,1 and PS 2. On the other hand, there was no significant difference in PFS and the results were almost equal, suggesting that PE-based regimen can be expected to have a good therapeutic outcome even if PS is not favorable.

**Table 5:** Prognostic analysis of PFS and OS from initiation of PE-based chemotherapy: univariate analysis.

Patients	PFS		OS	
	Median (M) [95%CI]	Log rank p-value	Median (M) [95%CI]	Log rank p-value
Age				
<65	4.4 [2.6-6.2]	0.49	14.3 [9.4-19.2]	0.21
≧65	5.2 [3.3-7.0]		21.4 [11.8-31.0]	
ECOG performance status score				
0, 1	5.3 [3.1-7.4]	0.58	18.6 [12.5-24.7]	0.0198
2	4.3 [0.0-6.6]		9.0 [4.9-13.1]	
Smoking status				
Current or former	4.6 [3.3-5.9]	0.25	18.6 [12.3-24.9]	0.65
Never	6.4 [1.6-11.3]		14.3 [13.4-15.1]	
PD-L1 status (CPS)				
1-19	4.4 [1.3-7.5]	0.94	17.1 [NA]	0.73
20-	5.3 [1.1-9.4]		NA [NA-NA]	
unknown	4.6 [2.0-7.2]		18.6 [11.8-25.5]	
Prior immunotherapy regimens				
Pembrolizumab	5.2 [3.3-7.0]	0.79	17.1 [NA]	0.43
Nivolumab	4.6 [2.0-7.2]		18.6 [11.8-25.5]	
Efficacy by ICI				
CR or PR or SD	6.4 [3.8-9.1]	0.51	18.7 [12.5-25.0]	0.21
PD	4.4 [3.4-5.4]		14.3 [8.8-19.7]	
Number of previous lines of systemic therapy before ICI for R/M SCCHN				
= 1^st^ line	5.3 [3.6-6.9]	0.029	18.6 [13.0-24.2]	0.18
2^nd^ line or more	4.3 [2.9-3.6]		12.5 [8.4-16.5]	
Regimen of PE-based chemo				
PCE	4.4 [2.8-6.0]	0.15	17.1 [10.5-23.7]	0.31
PTX+Cmab	5.7 [2.1-9.4]		18.6 [12.5-24.7]	

ECOG, Eastern Cooperative Oncology Group; CI, confidence interval; CPS, combined positive score; PFS, progression-free survival; ICI, immune check point inhibitor; CR, complete response; PR, partial response; SD, stable disease; PD, progression disease; PTX+Cmab, paclitaxel +cetuximab; PCE, paclitaxel+carboplatin+cetuximab.

**Figure 4:**
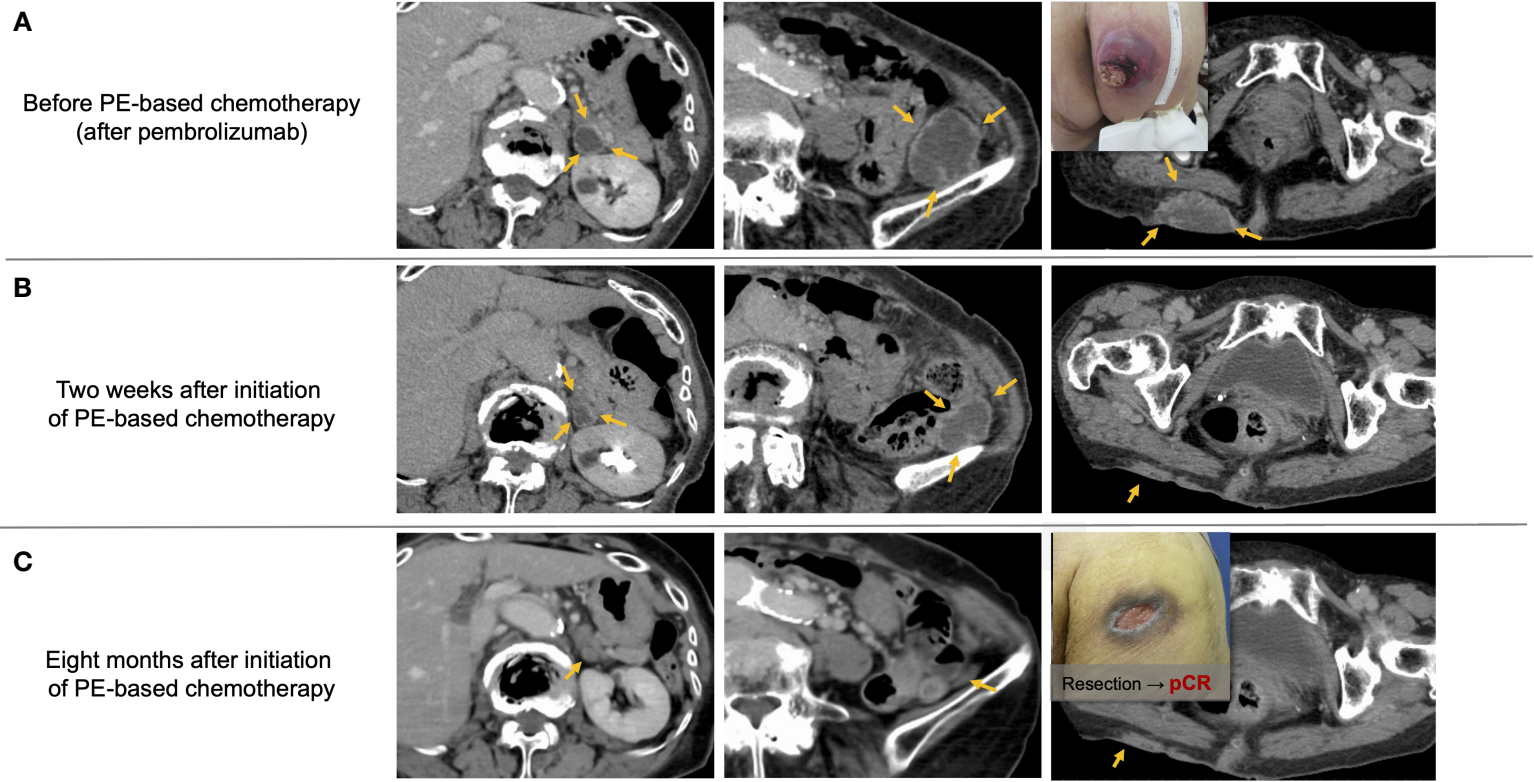
Representative images from a patient with recurrent oral gingival squamous cell carcinoma who achieved a favorable clinical response by PE-based chemotherapy after pembrolizumab failure. **(A)** Multiple subcutaneous and retroperitoneum tumors progressed after pembrolizumab treatment. **(B)** Tumor promptly responded to PCE therapy, and the response reached PR two weeks after the initiation of PCE. **(C)** Tumors almost disappeared, and the pathological complete response of the buttock tumor was proven by surgery for wound closure. PCE, paclitaxel+carboplatin+cetuximab; pCR, pathological complete response. Notes: PE-based chemotherapy, paclitaxel+carboplatin+cetuximab (PCE) or paclitaxel+cetuximab (PTX+Cmab).

**Figure 4** shows representative images from a patient with metastatic oral gingival squamous cell carcinoma who had a profound and durable tumor response by PCE. The patient experienced multiple subcutaneous and retroperitoneal metastases after initial surgery with marginal mandibulectomy and bilateral neck dissection (pT4aN2c, 16 months before recurrence) and additional neck dissection with adjuvant radiotherapy for late cervical lymph node recurrence (one month before recurrence). Pembrolizumab monotherapy was initiated as CPS in tissue newly obtained from a subcutaneous metastasis was around 50, and the patient had few tumor-related symptoms. However, the disease progressed within one week after the first administration, and subcutaneous metastasis on the hip ruptured and caused pain (**Figure 4A**). We immediately switched the patient to PCE and found that all recurrent lesions had macroscopically shrunk within a few days. Further, PR by RECIST criteria was identified by a CT scan taken on day 14 after PCE initiation (**Figure 4B**). Eventually, the patient obtained near-CR, and 25 months later is currently being treated with Cmab maintenance therapy. Further, pathological complete response of the buttock tumor with the presence of multinucleated giant cells was proven in surgery for wound closure (**Figure 4C**).

## Discussion

This study focused on PE-based chemotherapy as subsequent treatment following ICI in R/M SCCHN. Results showed a rapid (median time to response: 1.8 months) and favorable tumor response (ORR: 62.7%) irrespective of the efficacy of ICI, with a manageable safety profile. This study is the first detailed report of clinical outcomes with the therapeutic sequence of pembrolizumab followed by PCE in these patients.

Beginning even before the emergence of immunotherapy in clinical practice, PE-based chemotherapy was recognized as a treatment option for R/M SCCHN with encouraging survival outcomes as well as favorable tumor shrinkage. As in platinum-refractory cases, PE regimens have been widely used, and ORRs of 34-54% have been reported in patients without prior ICI therapy (6, 13–15). In contrast, several retrospective studies reported the more robust antitumor efficacy of PE-based chemotherapy (ORR; 41-70%) following ICI treatment (8, 10), probably due to favorable interaction between ICI and the regimen. In our study, the ORR with PE-based chemotherapy was also favorable, reaching 62.7%. In addition, two Phase II trials of ICI plus Cmab in R/M SCCHN have been reported, with response rates of 37% for pembrolizumab plus Cmab and 45% for pembrolizumab plus Cmab (16, 17). Considering the high response rate in this study, the combination of paclitaxel can be expected to add approximately 20% to the response rate. Notably, the favorable ORR values on combination of taxane plus Cmab in these studies, including our present study (56.8%), might obviate the need for clinical trials to evaluate the significance of adding Cmab to taxane, particularly as taxane plus Cmab is a widely accepted therapeutic option in this setting (11, 12). In this study, survival from the start of ICI was also very long (OSici: 23.3 months). This result is almost equivalent to the median OS of 21.9 months in the TPExtreme study, in which patients received cetuximab, docetaxel, and cisplatin followed by subsequent chemotherapy (18). On the other hand, the present results were longer than those of the median OS (14.9 months) in patients who received chemotherapy without ICI as a subsequent chemotherapy. These results suggest that immune checkpoint inhibitor therapy at any stage of the treatment sequence is important in the treatment of R/M SCCHN.

The PCE regimen has shown reliable efficacy primarily in platinum-sensitive settings. For example, the CSPOR HN02 study showed an ORR of 40.0% and median OS of 14.7 months in patients without prior immunotherapy. To date, however, the actual clinical efficacy of the PCE regimen following prior ICI therapy has not been reported in detail; accordingly, our present study is the first to provide reference data for clinically significant variables. Given that the KEYNOTE-048 study resulted in pembrolizumab monotherapy becoming a standard of care for patients with PD-L1-positive disease, and that subsequent taxane-containing therapy might maximize prognosis, we believe that PCE is appropriate for subsequent chemotherapy following pembrolizumab. Numerically speaking, the high tumor response (ORR of 80.0%, and 86.6% achieved at least a 30% reduction in the summed dimensions of target lesions, as shown in **Figure 1B**) and rapid tumor shrinkage presents a reliable strategy for overcoming the relatively low response rate with pembrolizumab monotherapy. Furthermore, the PFS of 5.2 months and OS of 17.1 months from the initiation of PE-based chemotherapy are also promising results. From this point, the observation that PE-based chemotherapy is effective even in cases where the effects of prior ICI treatment were insufficient is also a highly encouraging outcome for clinicians. In addition, the relatively low (40.7%) incidence of grade 3 or above AEs with PE-based chemotherapy is also notable. Again, given that other Cmab-containing chemotherapy regimens were associated with higher AE rates (e.g., 82% in the EXTREME regimen), the high tolerability of PE-based chemotherapy would benefit patients during treatment. Further, the favorable safety profile likely also contributes to the satisfactory transition rate to additional subsequent therapy following PE-based chemotherapy (82% in the current study), in turn eventually contributing to patient survival. A final benefit is that the PE-based chemotherapy can be provided on an outpatient basis, greatly benefitting this patient population, who still have a limited prognosis. While PE-based chemotherapy is generally easily given in clinics without hospitalization, other Cmab-containing chemotherapies, such as the EXTREME regimen, require hospital admission for 5-FU administration as well as hydration with cisplatin administration.

The study has several limitations. First, although we found that subsequent PE-based chemotherapy proved to be effective even in cases where ICI was not effective, prognostic analysis related to ICI treatment was not possible because only patients with disease progression on ICI were included in the study. Second, in the pembrolizumab cohort, no conclusive finding was obtained on the validity of choosing the PE-regimen over other Cmab-based chemotherapy (i.e., EXTREME regimen). Larger, background-matched comparisons or prospective trials may help answer this question.

## Conclusion

PE-based chemotherapy provides favorable and rapid antitumor efficacy in patients with R/M SCCHN as subsequent chemotherapy following ICI. It is therefore an encouraging treatment option in patients with R/M SCCHN who fail or do not respond to ICI Moreover, the high tolerability and outpatient availability of this regimen should greatly benefit this patient population.

## Data availability statement

The original contributions presented in the study are included in the article/**Supplementary Material**. Further inquiries can be directed to the corresponding author.

## Ethics statement

The studies involving humans were approved by National Cancer Center Hospital East Institutional Review Board. The studies were conducted in accordance with the local legislation and institutional requirements. The participants provided their written informed consent to participate in this study. Written informed consent was obtained from the individual(s) for the publication of any potentially identifiable images or data included in this article.

## Author contributions

HT participated in formulating the study concept and design, data curation, data interpretation, and drafting of the manuscript. TE participated in data interpretation and drafting of the manuscript. MT supervised the study and revised the manuscript. All authors contributed to the article and approved the submitted version.

## References

[1] Vermorken JB Mesia R Rivera F Remenar E Kawecki A Rottey S. Platinum-based chemotherapy plus cetuximab in head and neck cancer. N Engl J Med (2008) 359(11):1116–27. doi: 10.1056/NEJMoa0802656 18784101

[2] Ferris RL Blumenschein G Jr. Fayette J Guigay J Colevas AD Licitra L. Nivolumab for recurrent squamous-cell carcinoma of the head and neck. N Engl J Med (2016) 375(19):1856–67. doi: 10.1056/NEJMoa1602252 PMC5564292 27718784

[3] Burtness B Harrington KJ Greil R Soulières D Tahara M de Castro G Jr.. Pembrolizumab alone or with chemotherapy versus cetuximab with chemotherapy for recurrent or metastatic squamous cell carcinoma of the head and neck (KEYNOTE-048): a randomised, open-label, phase 3 study. Lancet (2019) 394(10212):1915–28. doi: 10.1016/S0140-6736(19)32591-7 31679945

[4] Harrington KJ Burtness B Greil R Soulières D Tahara M de Castro G Jr.. Pembrolizumab with or without chemotherapy in recurrent or metastatic head and neck squamous cell carcinoma: updated results of the phase III KEYNOTE-048 study. J Clin Oncol (2023) 41(4):790–802. doi: 10.1200/JCO.21.02508 36219809 PMC9902012

[5] Emens LA Middleton G. The interplay of immunotherapy and chemotherapy: harnessing potential synergies. Cancer Immunol Res (2015) 3(5):436–43. doi: 10.1158/2326-6066.CIR-15-0064 PMC5012642 25941355

[6] Enokida T Okano S Fujisawa T Ueda Y Uozumi S Tahara M. Paclitaxel plus cetuximab as 1st line chemotherapy in platinum-based chemoradiotherapy-refractory patients with squamous cell carcinoma of the head and neck. Front Oncol (2018) 8:339. doi: 10.3389/fonc.2018.00339 30211118 PMC6119881

[7] Tahara M Kiyota N Yokota T Hasegawa Y Muro K Takahashi S. Phase II trial of combination treatment with paclitaxel, carboplatin and cetuximab (PCE) as first-line treatment in patients with recurrent and/or metastatic squamous cell carcinoma of the head and neck (CSPOR-HN02). Ann Oncol (2018) 29(4):1004–9. doi: 10.1093/annonc/mdy040 29408977

[8] Minohara K Matoba T Kawakita D Takano G Oguri K Murashima A. Novel Prognostic Score for recurrent or metastatic head and neck cancer patients treated with Nivolumab. Sci Rep (2021) 11(1):16992. doi: 10.1038/s41598-021-96538-7 34417539 PMC8379150

[9] Sato Y Fukuda N Fujiwara YU Wang X Urasaki T Ohmoto A. Efficacy of paclitaxel-based chemotherapy after progression on nivolumab for head and neck cancer. In Vivo (2021) 35(2):1211–5. doi: 10.21873/invivo.12371 PMC8045096 33622923

[10] Wakasaki T Yasumatsu R Uchi R Taura M Matsuo M Komune N. Outcome of chemotherapy following nivolumab treatment for recurrent and/or metastatic head and neck squamous cell carcinoma. Auris Nasus Larynx (2020) 47(1):116–22. doi: 10.1016/j.anl.2019.05.001 31128940

[11] Billan S Kaidar-Person O Gil Z. Treatment after progression in the era of immunotherapy. Lancet Oncol (2020) 21(10):e463–e76. doi: 10.1016/S1470-2045(20)30328-4 33002442

[12] Saba NF Chen ZG Haigentz M Bossi P Rinaldo A Rodrigo JP. Targeting the EGFR and immune pathways in squamous cell carcinoma of the head and neck (SCCHN): forging a new alliance. Mol Cancer Ther (2019) 18(11):1909–15. doi: 10.1158/1535-7163.MCT-19-0214 PMC6830522 31676542

[13] Hitt R Irigoyen A Cortes-Funes H Grau JJ García-Sáenz JA Cruz-Hernandez JJ. Phase II study of the combination of cetuximab and weekly paclitaxel in the first-line treatment of patients with recurrent and/or metastatic squamous cell carcinoma of head and neck. Ann Oncol (2012) 23(4):1016–22. doi: 10.1093/annonc/mdr367 21865152

[14] Motai R Sawabe M Kadowaki S Sasaki E Nishikawa D Suzuki H. Clinical impact of weekly paclitaxel plus cetuximab is comparable to the EXTREME regimen for recurrent/metastatic head and neck squamous cell carcinoma. Int J Clin Oncol (2021) 26(7):1188–95. doi: 10.1007/s10147-021-01907-x 33821363

[15] Okada T Okamoto I Sato H Ito T Miyake K Tsukahara K. Efficacy and safety of paclitaxel combined with cetuximab for head and neck squamous cell carcinoma. In Vivo (2021) 35(2):1253–9. doi: 10.21873/invivo.12376 PMC8045122 33622928

[16] Chung CH Li J Steuer CE Bhateja P Johnson M Masannat J. Phase II multi-institutional clinical trial result of concurrent cetuximab and nivolumab in recurrent and/or metastatic head and neck squamous cell carcinoma. Clin Cancer Res (2022) 28(11):2329–38. doi: 10.1158/1078-0432.CCR-21-3849 PMC9167762 35344035

[17] Sacco AG Chen R Worden FP Wong DJL Adkins D Swiecicki P. Pembrolizumab plus cetuximab in patients with recurrent or metastatic head and neck squamous cell carcinoma: an open-label, multi-arm, non-randomised, multicentre, phase 2 trial. Lancet Oncol (2021) 22(6):883–92. doi: 10.1016/S1470-2045(21)00136-4 PMC12140401 33989559

[18] Guigay J Aupérin A Fayette J Saada-Bouzid E Lafond C Taberna M. Cetuximab, docetaxel, and cisplatin versus platinum, fluorouracil, and cetuximab as first-line treatment in patients with recurrent or metastatic head and neck squamous-cell carcinoma (GORTEC 2014-01 TPExtreme): a multicentre, open-label, randomised, phase 2 trial. Lancet Oncol (2021) 22(4):463–75. doi: 10.1016/S1470-2045(20)30755-5 33684370

