# Supplementary material for: Subsequent chemotherapy with paclitaxel plus cetuximab-based chemotherapy following immune checkpoint inhibitor in recurrent or metastatic squamous cell carcinoma of the head and neck
Source: Front Oncol. 2023 Nov 21;13:1221352. doi: 10.3389/fonc.2023.1221352 (PMC10703151; doi:10.3389/fonc.2023.1221352)
Supplement: Supplementary file 1 [file DataSheet_1.docx]

# Supplementary Figures and Tables

**Supplementary Figure 1.** Patient flow diagram

(A) Patient flow diagram of the pembrolizumab cohort

**
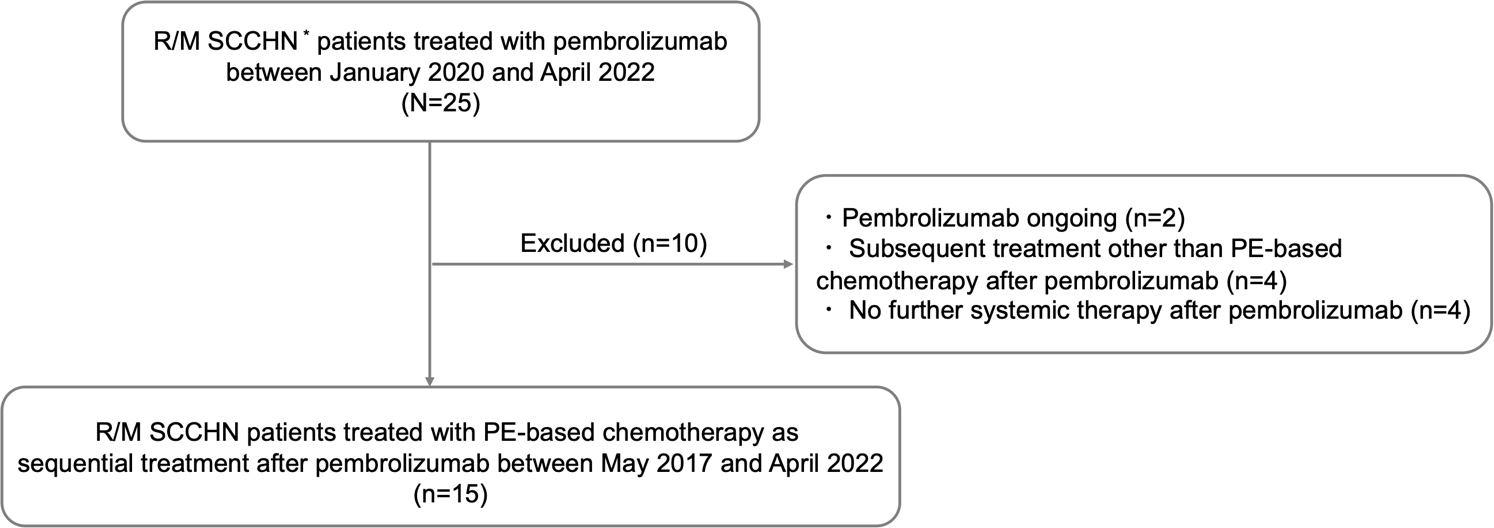
**

(B) Patient flow diagram of the nivolumab cohort


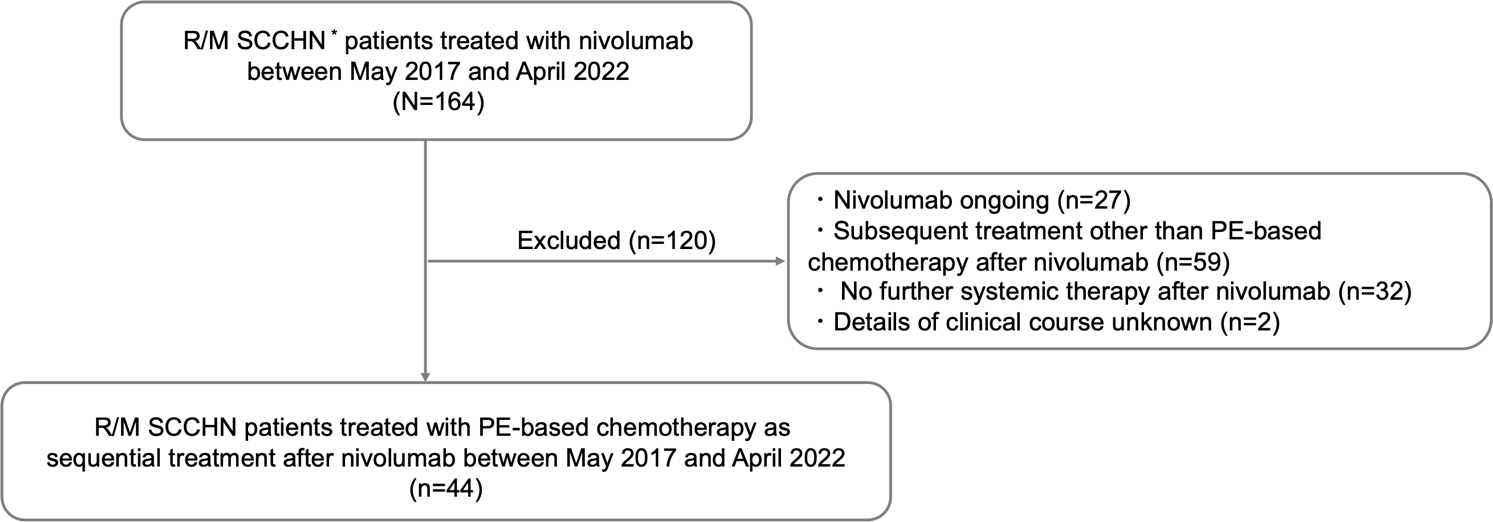


Abbreviations: R/M SCCHN, recurrent/metastatic squamous cell carcinoma of the head and neck. Note: *Primary site, including oral cavity, oropharynx, hypopharynx, and larynx; PE-based chemotherapy, paclitaxel+carboplatin+cetuximab (PCE) or paclitaxel+cetuximab (PTX+Cmab).

## Supplementary Figure 2. Correlation of response between PE-based chemotherapy and ICI monotherapy


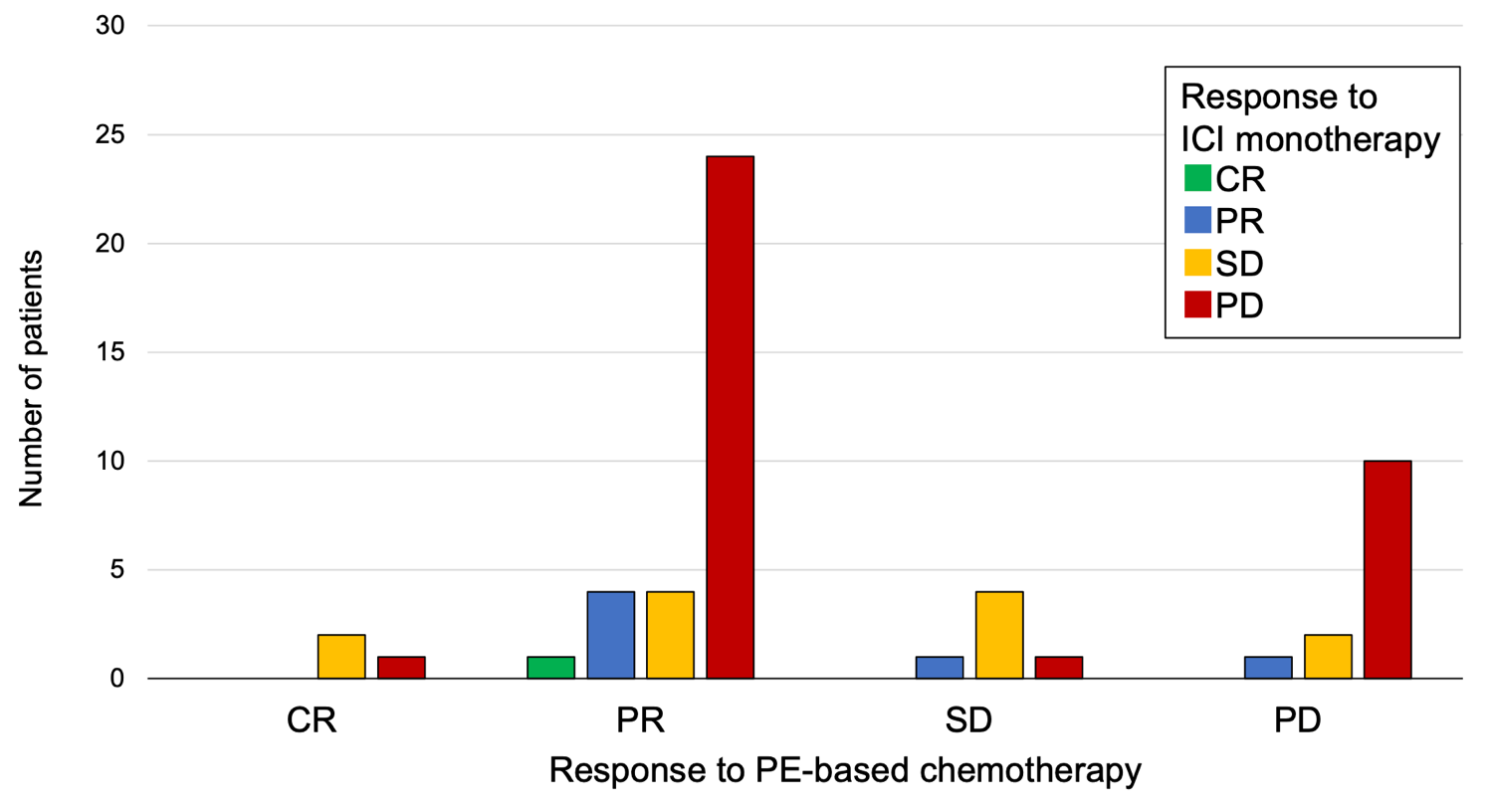


## Abbreviations: CR, complete response; PR, partial response; SD, stable disease; PD, progressive disease; ICI, immune checkpoint inhibitor. Note: PE-based chemotherapy, paclitaxel+carboplatin+cetuximab (PCE) or paclitaxel+cetuximab (PTX+Cmab).

## Supplementary Figure 3. Treatment delivery after ICI

##
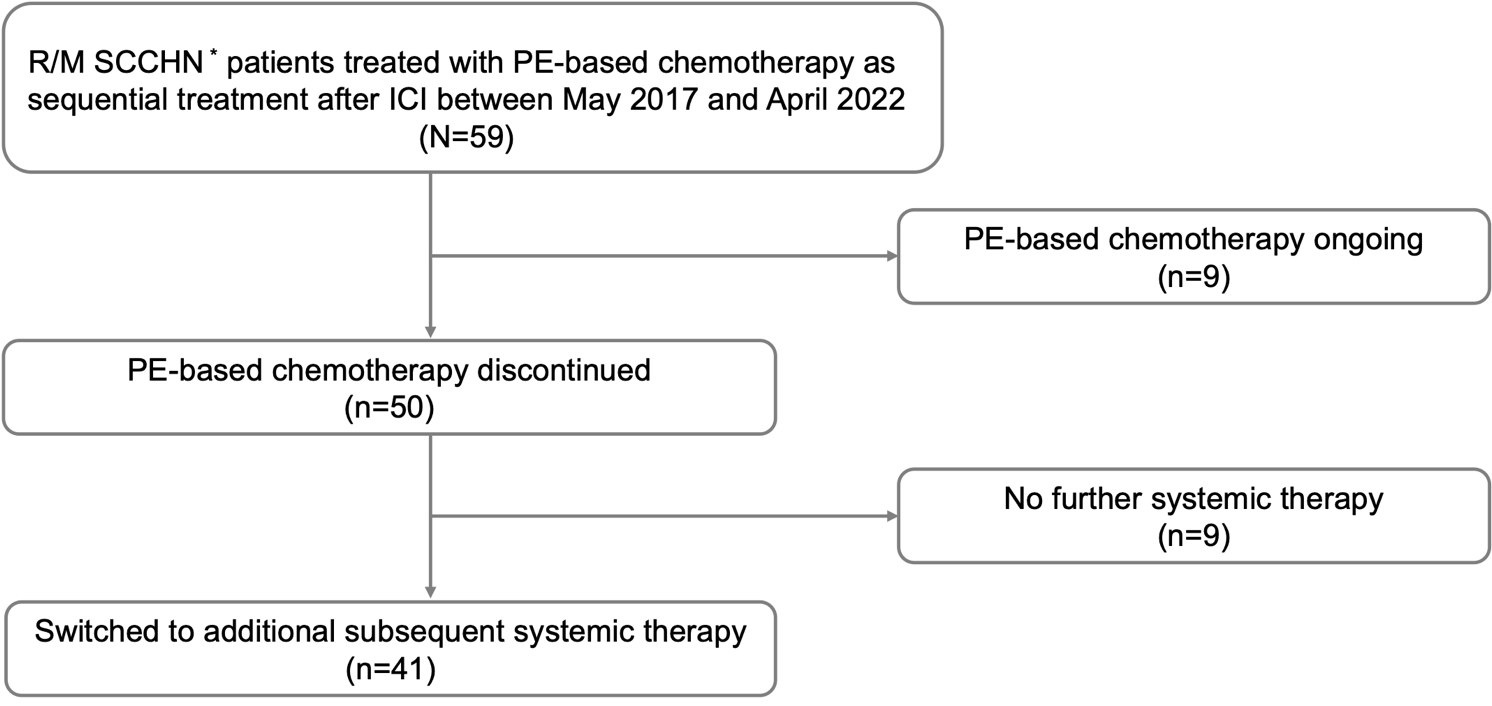


Abbreviations: mPFS, median progression-free survival; CI, confidence interval; HR, hazard ratio. Note: *Primary site, including oral cavity, oropharynx, hypopharynx, and larynx; PE-based chemotherapy, paclitaxel+carboplatin+cetuximab (PCE) or paclitaxel+cetuximab (PTX+Cmab).

**Supplementary Figure 4.** Comparison of PFS and OS in Subsequent Chemotherapy after ICI

: PE-based chemotherapy vs. Other regimens.


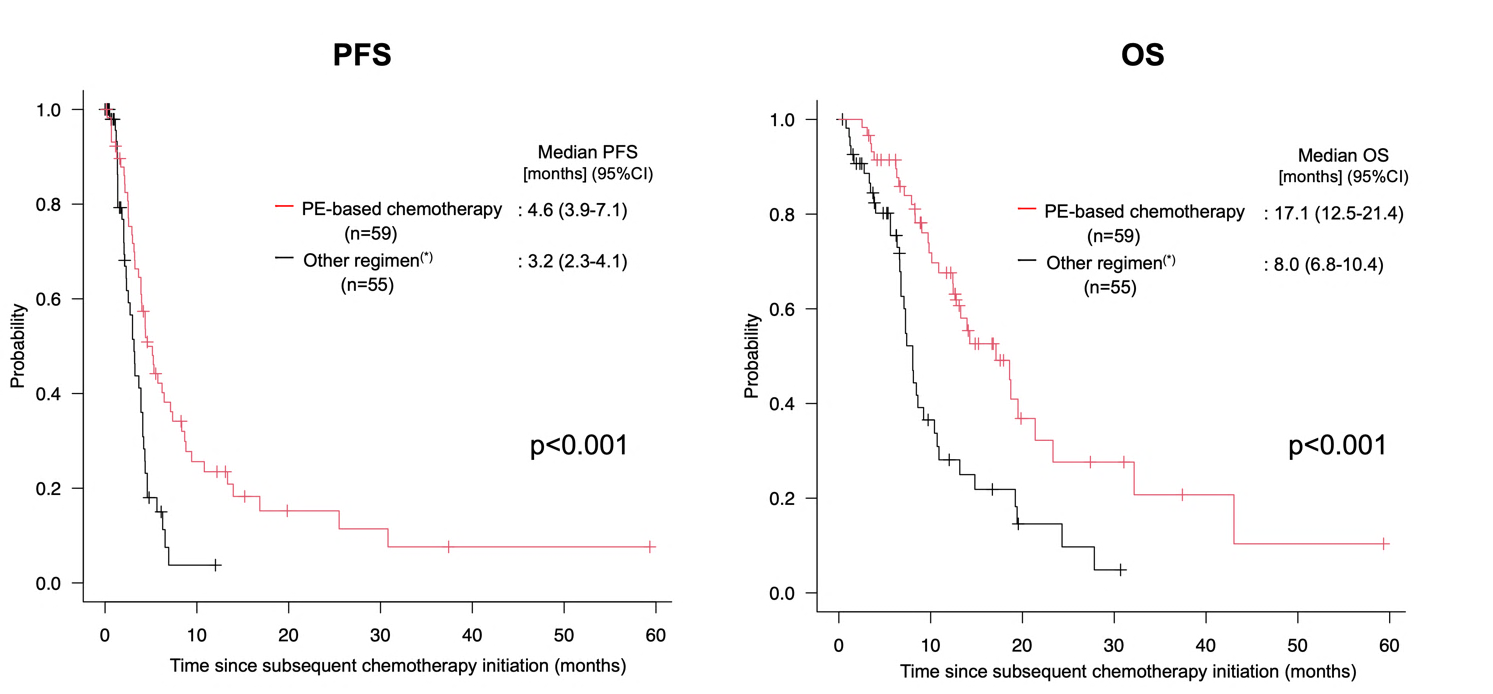


Abbreviations: PFS, progression-free survival; CI, confidence interval; OS, overall survival. Note: (*)Other regimen contains S-1 (n=40), paclitaxel (n=12), carboplatin+paclitaxel (n=2), cisplatin+5-FU+cetuximab (n=1)

**Supplementary Figure 5.** PFS, OS, PFS2 and OSici of the patients treated with pembrolizumab as prior ICI.

A, B: Progression-free survival (PFS) and overall survival (OS) from the initiation of PE-based regimen


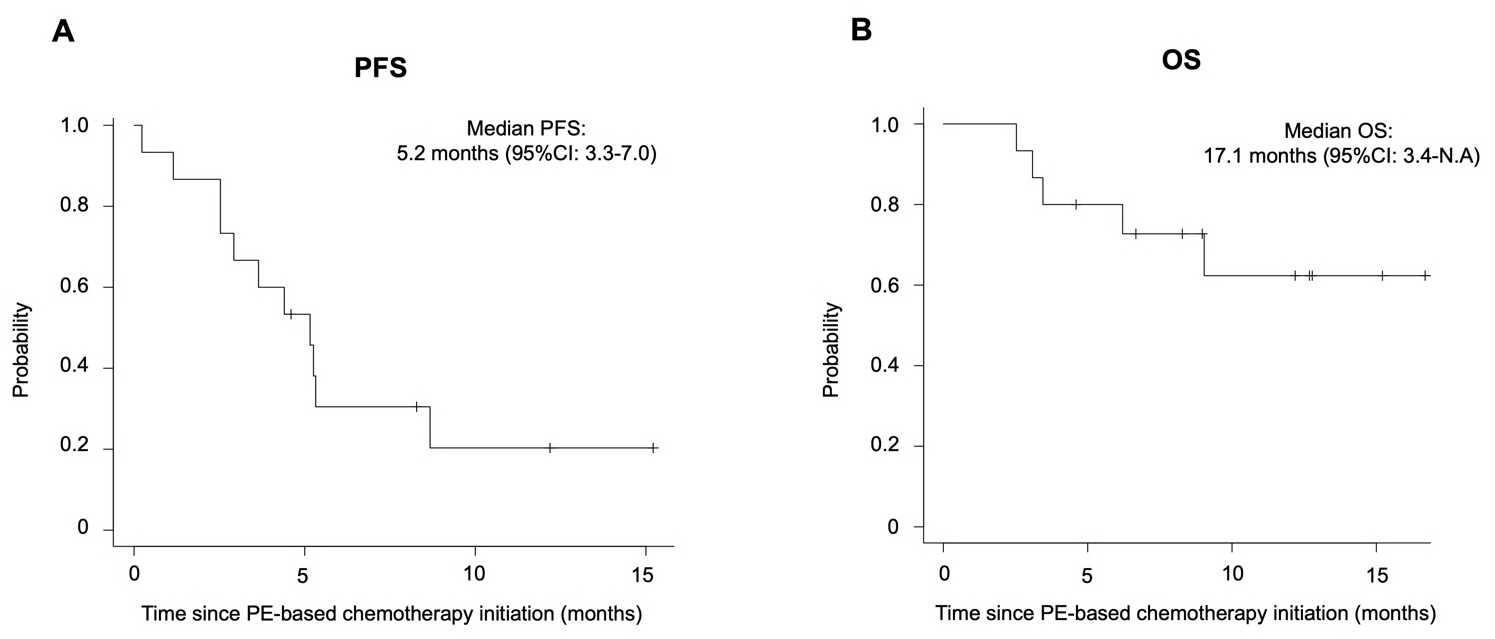


C, D: Progression-free survival 2 (PFS2) (C) and overall survival (OSici) (D) from the initiation of pembrolizumab monotherapy
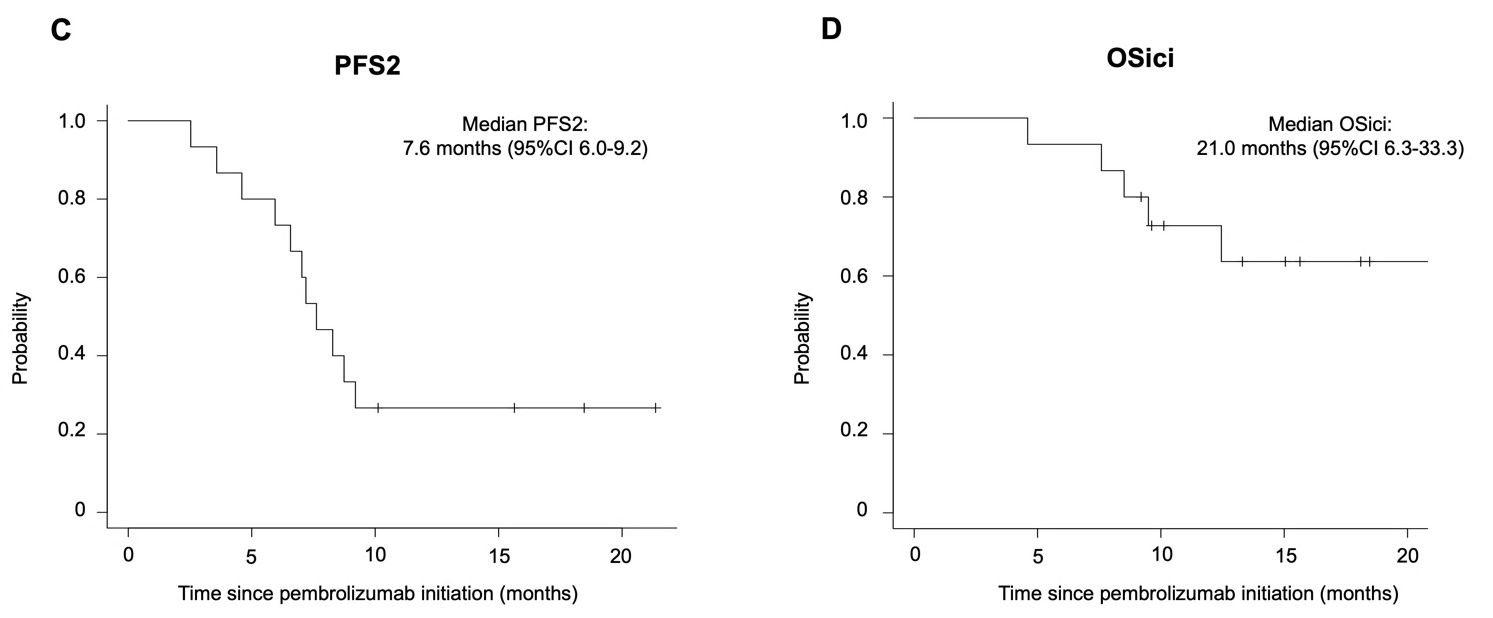


Abbreviations: PFS, progression-free survival; PFS2, progression-free survival 2; OS, overall survival; CI, confidence interval; N.A, not available; ICI, Immune checkpoint inhibitor. Notes: OSici, overall survival from initiation of immune checkpoint inhibitors.

**Supplementary Figure 6.** PFS, OS, PFS2 and OSici of the patients treated with nivolumab as prior ICI.

A, B: Progression-free survival (PFS)(A) and overall survival (OS) (B)from the initiation of PE-based regimen
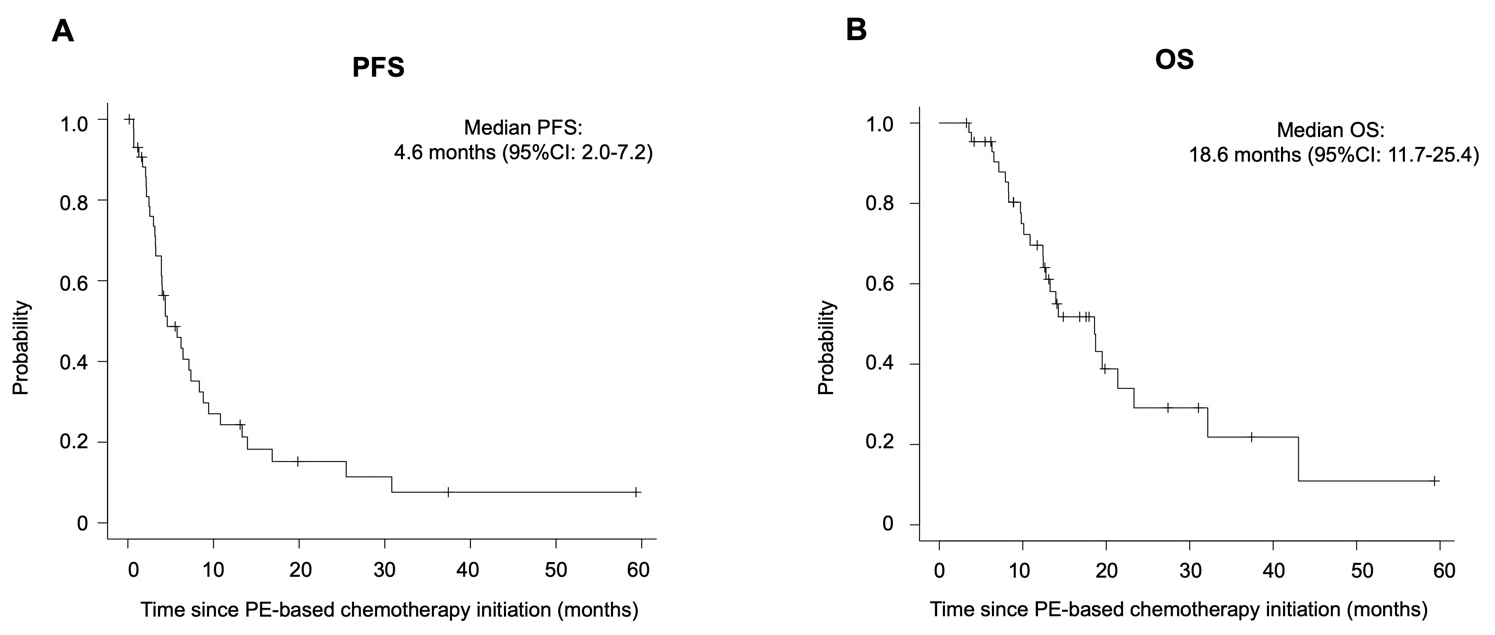


C, D: Progression-free survival 2(PFS2) (C) and overall survival (OSici) (D) from the initiation of nivolumab


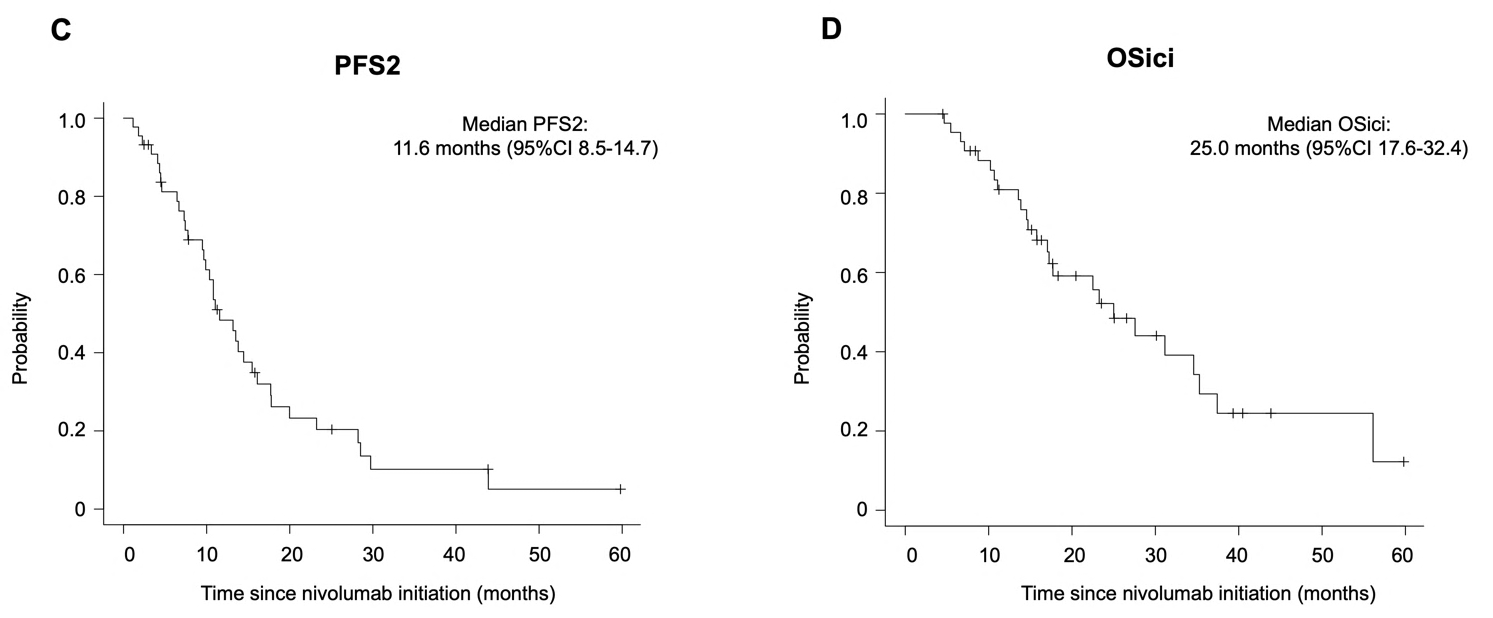


Abbreviations: PFS, progression-free survival; PFS2, progression-free survival 2; OS, overall survival; CI, confidence interval; N.A, not available; ICI, Immune checkpoint inhibitor. Notes: OSici, overall survival from initiation of immune checkpoint inhibitors.**Supplementary Figure 7. Outcome of PE-based regimen focusing on platinum sensitivity**

A, B: Response of patients treated with a PE-based regimen following ICI monotherapy


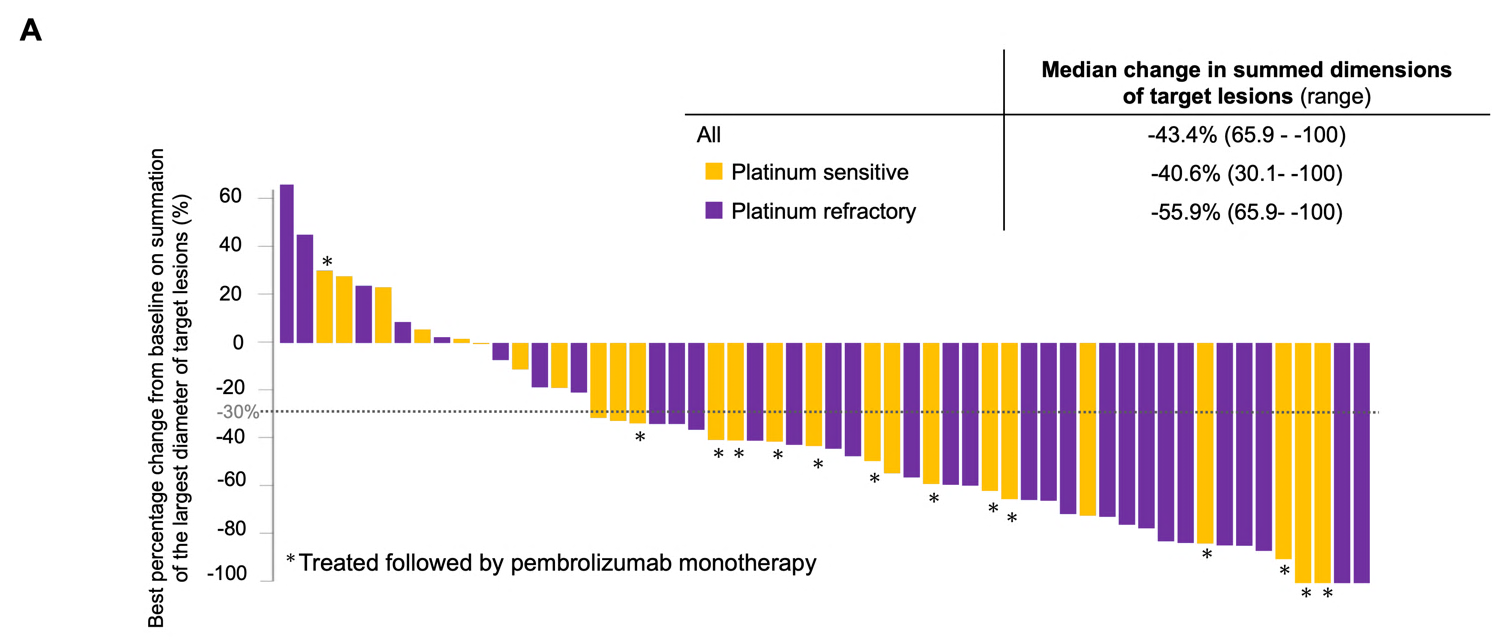

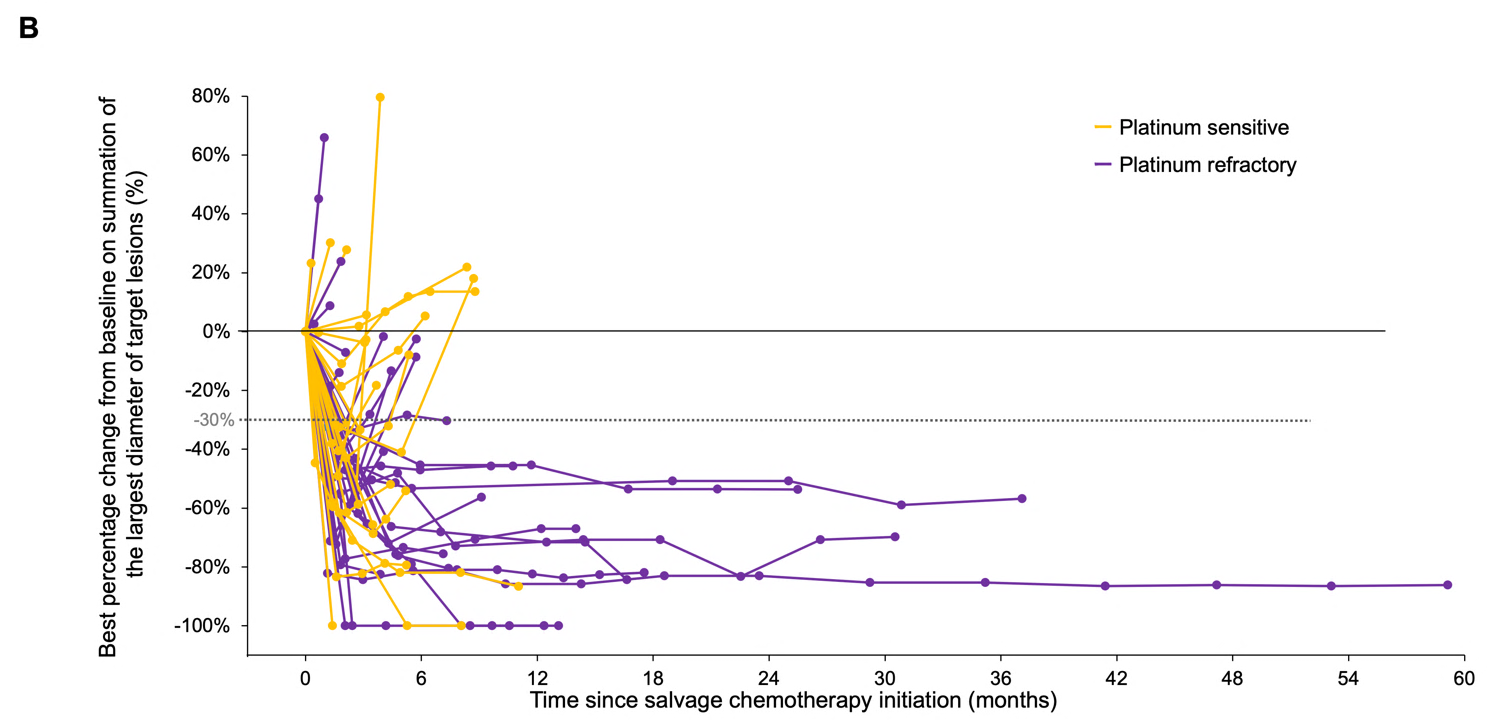


C, D: Progression-free survival (PFS)(C)and overall survival (OS)(D) from the initiation of PE-based regimen
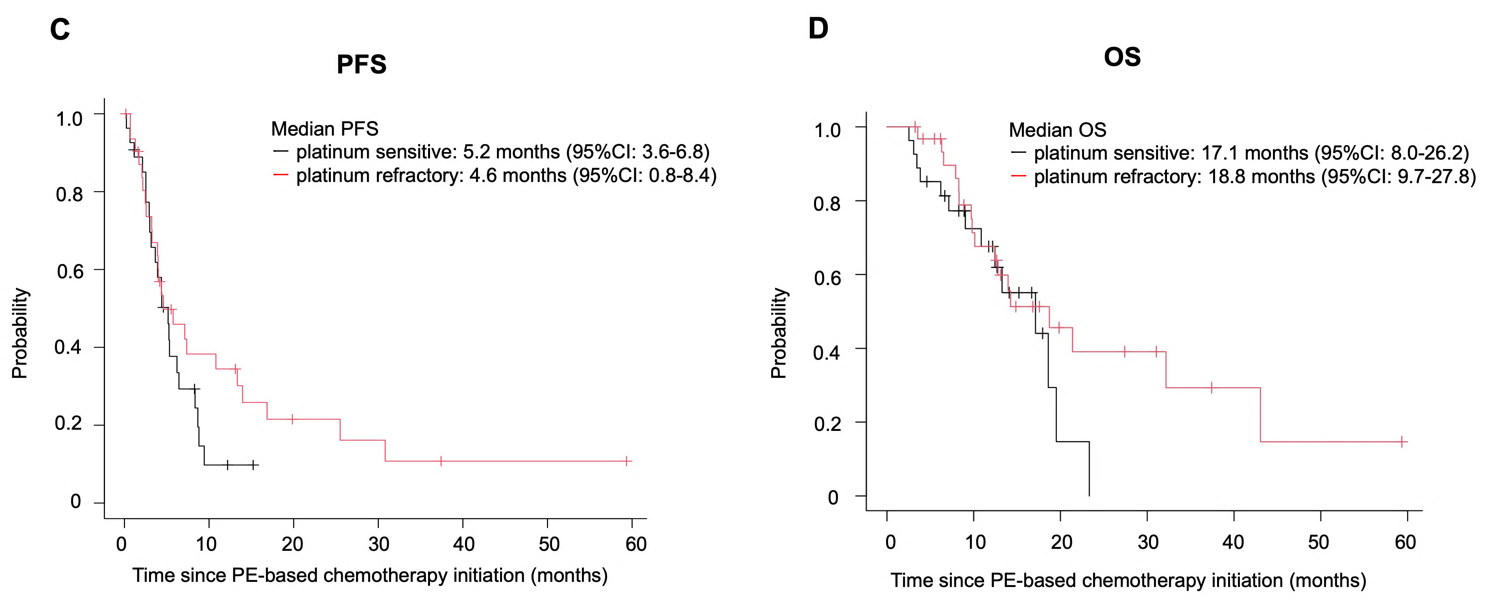


E, F: Progression-free survival 2(PFS2) (E) and overall survival (OSici) (F) from the initiation of ICI
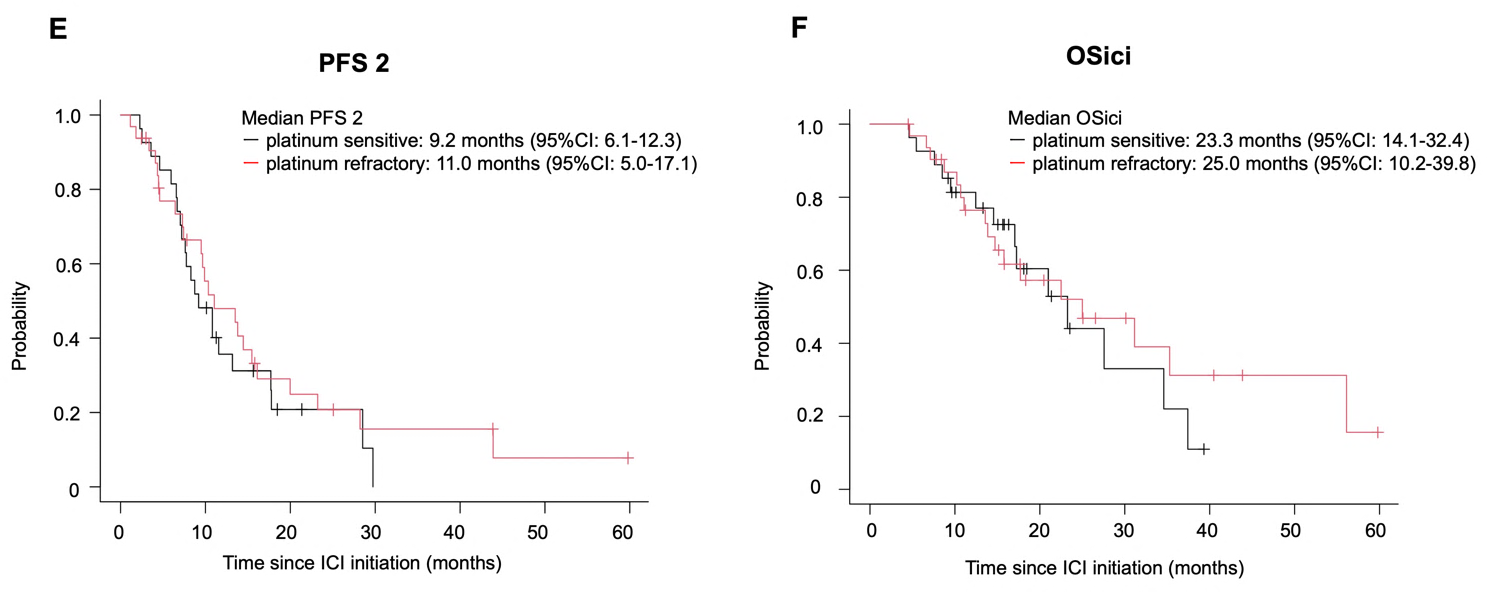


Abbreviations: PFS, progression-free survival; OS, overall survival; PFS2, progression-free survival 2; CI, confidence interval. Note: PE-based chemotherapy, PE-based chemotherapy, paclitaxel+carboplatin+cetuximab (PCE) or paclitaxel+cetuximab (PTX+Cmab); ICI, pembrolizumab or nivolumab; OSici, overall survival from initiation of prior immune checkpoint inhibitor; Platinum-sensitive was defined as disease progression or recurrence after 6 months from the end of platinum chemotherapy, and platinum-refractory was defined as occurring within 6 months.

**Supplementary Table 1.** Patient characteristics (pembrolizumab vs. nivolumab)

| **Characteristic** | **Number of patients**, (%) | | **P-value** |
| --- | --- | --- | --- |
|  | **Pembrolizumab (n=15)** | **Nivolumab (n=44)** |  |
| **Median Age**, years [range] | 65 [41-79] | 62.5 [19-74] | 0.43 |
| **Gender**  　Male  　Female | 13 (86.7)  2 (13.3) | 33 (75)  11 (25) | 0.35 |
| **ECOG performance status score**  　0  　1  　≥2  Unknown | 8 (53.3)  5 (33.3)  2 (13.3)  0 (0) | 19 (43.2)  17 (38.6)  4 (9.1)  4 (9.1) | 0.59 |
| **Primary tumor site**  Oral cavity  Hypopharynx  Larynx  Oropharynx  p16 (+)  p16 (-)  p16 unknown | 5 (33.3)  2 (13.3)  2 (13.3)  6 (40)  2 (33.3)  3 (50)  1 (16.7) | 17 (38.6)  11 (25)  3 (6.8)  13 (29.5)  4 (30.8)  4 (30.8)  5 (38.5) | 0.63 |
| **Smoking status**  　Current or former  　Never | 13 (86.7)  2 (13.3) | 35 (79.5)  9 (20.5) | 0.54 |
| **Disease distribution**  　Locoregional only  　Distant only  　Local/regional and distant | 7 (46.7)  5 (33.3)  3 (20) | 18 (40.9)  14 (31.8)  12 (27.3) | 0.85 |
| **Platinum sensitivity^*^**  　Sensitive  　Refractory | 15 (100)^**^  0 (0) | 12 (27.3)  32 (72.7) | <0.01 |
| **Efficacy by ICI**  Complete response (CR)  Partial response (PR)  Stable disease  Progressive disease  Not evaluable | 0 (0)  2 (13.3)  3 (20)  10 (66.7)  0 (0) | 1 (2.3)  4 (9.1)  9 (20.5)  28 (63.6)  2 (4.5) | 0.91 |
| **ORR by prior ICI** (%) | 13.3 | 11.4 |  |
| **Median PFS by prior ICI**, month [95%CI] | 2.0 [0.6-2.2] | 3.2 [1.8-4.6] | 0.43 |
| **Number of previous lines of systemic**  **therapy before ICI for R/M SCCHN**  1  2  ≥3 | 15 (100)  0 (0)  0 (0) | 33 (75)  10 (22.7)  1 (2.3) | 0.10 |
| **Regimen of PE-based chemotherapy**  PCE  PTX+Cmab | 11 (73.3)  4 (26.7) | 8 (18.2)  36 (81.8) | <0.01 |

Abbreviations: ECOG, Eastern Cooperative Oncology Group; CI, confidence interval; ORR, objective response rate; PFS, progression-free survival; ICI; immune check point inhibitor; R/M HNSCC, recurrent/metastatic squamous cell carcinoma of the head and neck; PTX+Cmab, paclitaxel and cetuximab; PCE, paclitaxel+carboplatin+cetuximab. Note: Platinum-sensitive was defined as disease progression or recurrence after 6 months from the end of platinum chemotherapy, and platinum-refractory was defined as occurring within 6 months; ORR, proportion of CR+PR.

**Supplementary Table 2.** Patient characteristics and outcomes (platinum sensitivity)

| **Characteristic** | **Number of patients**, (%) | | **P-value** |
| --- | --- | --- | --- |
|  | **Platinum sensitive (n=27)** | **Platinum refractory (n=32)** |  |
| **Median Age**, years [range] | 60.4 [41-79] | 58.2 [19-73] | 0.52 |
| **Gender**  　Male  　Female | 19 (70.4)  8 (29.6) | 27 (84.4)  5 (15.6) | 0.22 |
| **ECOG performance status score**  　0  　1  　2  Unknown | 16 (59.3)  8 (29.6)  2 (7.4)  1 (3.7) | 11 (34.4)  14(43.8)  4 (12.5)  3 (9.4) | 0.303 |
| **Primary tumor site**  Oral cavity  Hypopharynx  Larynx  Oropharynx  p16 (+)  p16 (-)  p16 unknown | 8 (29.6)  5 (18.5)  2 (7.4)  12 (44.4)  5 (41.7)  4 (33.3)  3 (25.0) | 14 (43.8)  8 (25.0)  3 (9.4)  7 (21.9)  1 (14.3)  3 (42.9)  3 (42.9) | 0.63 |
| **Smoking status**  　Current or former  　Never | 23 (85.2)  4 (14.8) | 25 (78.1)  7 (21.9) | 0.54 |
| **Disease distribution**  　Locoregional only  　Distant only  　Local/regional and distant | 16 (59.3)  7 (25.9)  4 (14.8) | 9 (28.1)  12 (37.5)  11 (34.4) | 0.054 |
| **Prior immunotherapy regimens**  Pembrolizumab  Nivolumab | 15 (55.6)  12 (44.4) | 0 (0)  32 (100) | <0.01 |
| **Efficacy by ICI**  Complete response (CR)  Partial response (PR)  Stable disease  Progressive disease  Not evaluable | 0 (0.0)  4 (14.8)  7 (25.9)  15 (55.6)  1 (3.7) | 1 (3.1)  2 (6.2)  5 (15.6)  23 (71.9)  1 (3.1) | 0.52 |
| **ORR by prior ICI,** % | 14.8 | 9.3 |  |
| **Median PFS by prior ICI**, month [95%CI] | 3.4 [0.9-6.0] | 2.1 [0.9-3.0] | 0.12 |
| **Number of previous lines of systemic**  **therapy before ICI for R/M SCCHN**  1  2  ≥3 | 18 (66.7)  8 (29.6)  1 (3.7) | 30 (93.8)  2 (6.2)  0 (0.0) | 0.022 |
| **Regimen of PE-based chemotherapy**  PCE  PTX+Cmab | 19 (70.4)  8 (29.6) | 0 (0.0)  32 (100) | <0.01 |
| **Efficacy by PE-based chemotherapy**  Complete response (CR)  Partial response (PR)  Stable disease  Progressive disease  Not evaluable | 2 (7.4)  14 (51.9)  5 (18.5)  5 (18.5)  1 (3.7) | 1 (3.1)  20 (62.5)  2 (6.3)  8 (25)  1 (3.1) | 0.56 |
| **Median PFS, month [95%CI]** | 5.2 [3.6-6.8] | 4.6 [0.8-8.4] | 0.19 |
| **Median OS, month [95%CI]** | 17.1 [8.0-26.2] | 18.7 [9.7-27.8] | 0.25 |
| **Median PFS2, month [95%CI]** | 9.2 [6.1-12.3] | 11.0 [5.0-17.1] | 0.50 |
| **Median OSici, , month [95%CI]** | 23.3 [14.1-32.4] | 25.0 [10.2-39.8] | 0.63 |

Abbreviations: ECOG, Eastern Cooperative Oncology Group; CI, confidence interval; ORR, objective response rate; PFS, progression-free survival; ICI; immune check point inhibitor; R/M HNSCC, recurrent/metastatic squamous cell carcinoma of the head and neck; PE, paclitaxel and cetuximab; PCE, paclitaxel+carboplatin+cetuximab. Note: * Platinum-sensitive was defined as disease progression or recurrence after 6 months from the end of platinum chemotherapy, and platinum-refractory was defined as occurring within 6 months; **All patients in the pembrolizumab cohort harbored PD-L1-positive disease; ORR, proportion of CR+PR.
